# The composition of energy flow in the upper extremity under different lower extremity mechanics in baseball pitching

**DOI:** 10.1371/journal.pone.0361030

**Published:** 2026-10-23

**Authors:** Ryota Matsuda, Yuichi Hirano, Jun Umakoshi, Arata Kimura

**Affiliations:** 1 Graduate School of Sports and Health Studies, Hosei University, Tokyo, Japan; 2 Department of Sports and Health, Faculty of Sports and Health Studies, Hosei University, Tokyo, Japan; Instituto Politecnico de Viana do Castelo, PORTUGAL

## Abstract

Generating high ball speed is crucial for achieving competitive baseball pitching performance. The lower extremity mechanics play a key role in generating high ball speed during a baseball pitch. However, some previous studies have reported no significant difference in ball speeds despite differences in lower extremity mechanics, highlighting the need to examine how energy is transferred through the upper extremity to the ball under these conditions. This study examined how upper extremity energy flow differed across stride-length conditions in relation to the observed differences in ball speed. Twenty college baseball pitchers pitched with under-stride length (US) and over-stride length (OS) in addition to normal-stride length (NS). We analyzed the mechanical energy of the upper extremity segments at key events during pitching, and the energy flow through each joint between events. The mechanical energy in the proximal segments at the stride foot contact increased as the stride length extended. In contrast, the mechanical energy in the distal segments at maximum external rotation in the shoulder joint was greater in the NS than in the US and OS, consistent with ball speed differences. During this phase, the energy flow to the upper arm through the shoulder joint was greater in the NS than in the US and OS, and the same condition pattern was observed for energy transfer through the elbow and wrist joints and for ball speed. Additionally, although no statistically significant difference in ball speed was observed between the US and OS, the JFP and STP components of shoulder energy flow showed different condition patterns, suggesting that shoulder energy flow composition may differ even when no statistically significant difference is observed in total energy flow into the upper arm.

## Introduction

Generating high ball speed is crucial for achieving competitive baseball pitching performance. The pitcher first elevates the stride leg, moves forward toward home plate, plants the stride foot, and rapidly rotates the trunk to release the ball. This motion follows a proximal-to-distal sequence of whole-body movements commonly referred to as a kinetic chain, in which mechanical linkages between successive body segments transfer energy from proximal to distal segments. Numerous studies have focused on lower extremity movement [1–7] because lower extremities play a key role in generating and initiating energy flow in the kinetic chain during baseball pitching [8,9].

Stride length is a significant parameter in lower extremity mechanics for achieving high ball speed. Several cross-sectional studies indicated a significant correlation between the stride length and ball speed [10,11], and some suggested that the stride length can predict ball speed [12,13]. Collectively, these findings support an association between longer stride length and higher ball speed. However, some studies focusing on experimentally altered stride lengths did not support this finding [14,15]. Ramsey et al. (2014) showed that there was no significant difference in ball speed between the under-stride and over-stride lengths, despite the total body linear momentum in the throwing direction increasing with the over-stride length. They further showed that the under-stride length allowed for an increase in the rotational momentum in the transverse plane compared to that of the over-stride length [15]. These findings imply that altering stride length changes the pitching mechanics but results in no significant difference in ball speed. However, it is difficult to fully explain ball speed based on whole-body linear or rotational momentum in pitching, which involves a large number of degrees of freedom. Therefore, a more detailed mechanical perspective is required to understand why no significant difference in ball speed is generated despite changes in the stride length.

Baseball pitching involves the transfer of mechanical energy through the kinetic chain toward the ball from an energetic perspective [16,17]. Therefore, investigating how energy is transferred to the hand, which releases the ball, may provide insight into why no significant difference in ball speed is achieved. In whole-body pitching, the lower and upper extremities play distinct roles within the kinetic chain. The lower extremities primarily generate mechanical energy [4], whereas the upper extremity mainly transfers energy to the distal segments [16,18]. Accordingly, two possible mechanisms may explain the generation of no significant difference in ball speed under different stride length conditions. One possibility is that energy generated by the lower extremities is transferred through the upper extremity in a similar manner across conditions. In contrast, differences in energy generation at the lower extremities may be compensated for by differences in energy transfer within the upper extremity, resulting in a comparable amount of energy ultimately delivered to the ball.

Some studies investigated energy generation in the lower extremities across different stride length conditions. Previous studies have shown that altering stride length changes ground reaction force and energy generation in the lower extremities [19,20]. However, these changes do not necessarily result in differences in the amount of energy flow to the upper torso [19]. No significant difference in ball speed would be expected across different stride length conditions if energy transfer through the upper extremity is preserved. Interestingly, a previous study that examined three conditions, including the normal-stride length, reported that ball speed was higher at the normal-stride length than at the over-stride and under-stride lengths. Based on these results, it can be inferred that the manner of energy transfer at the upper extremity changes across stride length conditions and may play an important role in determining ball speed, although the amount of energy flowing to the upper torso is not significantly different.

Some studies reported the importance of energy flow in the upper extremity for generating high ball speed in whole-body pitching mechanics [16,17]. These studies showed significant correlations between ball speed and energy flow at the shoulder, elbow, and wrist joints. However, the mechanisms of energy flow differ across these joints. The elbow and wrist joints generate little energy and transfer it, whereas the shoulder joint generates and transfers energy [16,21]. Therefore, Köhler and Witt (2023) suggested that the shoulder joint is the last joint adding energy into the arm in throwing movements.

Accordingly, the two general possibilities described above can be examined by identifying where condition differences in energy flow emerge within the upper extremity. The first possibility is that the amount of energy flow to the arm through the shoulder joint is different, resulting in a different amount of energy being transferred to the ball through the elbow and wrist joints. The second possibility is that the amount of energy flow to the arm through the shoulder joint is similar, but the energy transfer through the elbow and wrist joints is different, leading to a different amount of energy ultimately delivered to the ball. However, no studies have investigated the energy flow at the upper extremity under no significant difference in energy flow to the upper torso, and it remains unclear which possibility is engaged during pitching.

Investigating the energy flow at the upper extremity would enhance understanding of why different ball speeds are generated despite no significant difference in energy flow to the upper torso. Furthermore, this investigation may help explain the central question in baseball biomechanics, which is how pitchers achieve high ball speed. Therefore, the purpose of this study was to examine how upper extremity energy flow differed across stride length conditions in relation to the observed differences in ball speed. Previous findings showed no statistically significant differences in energy flow to the upper torso across stride-length conditions [19]. Accordingly, we hypothesized that altering stride length would influence energy flow within the upper extremity during pitching. Specifically, we expected that differences in stride length would be reflected either in the magnitude of energy flow to the upper arm through the shoulder joint or in the way energy is transferred through the distal joints, resulting in different ball speeds.

## Methods

Data used in this study were obtained from a previously conducted experiment on baseball pitching under different stride length conditions [19]. The original study examined how altering stride length affects energy flow in the lower extremities, which primarily generate mechanical energy. In contrast, the present study reanalyzes the same dataset to investigate how energy is transferred through the upper extremity to the ball.

### Participants

Participants were recruited between 01/04/2023 and 31/08/2023. A total of twenty male college baseball pitchers (age: 19.9 ± 1.1 years; body height: 173.2 ± 5.8 cm; body mass: 71.8 ± 6.4 kg) volunteered to participate in this study. All participants met the following criteria. 1) Participants were overarm or three-quarter-arm throwers, and not side-arm or underarm throwers. 2) Participants had no injuries. 3) Participants were able to pitch with maximal effort. Prior to participation, all individuals received a detailed explanation of this study’s procedures and provided written informed consent. The study protocol complied with the Declaration of Helsinki and was approved by the Ethical Committee of the Graduate School of Sports and Health Studies at Hosei University (approval number: 2022_26).

All 20 participants and all trials included in the original study were retained in the present analysis, with no additional participant or trial exclusions. None of the upper-extremity energy-flow variables planned for analysis were omitted. The upper-extremity energy-flow analysis was planned before the results were examined. Because the present study was a planned secondary analysis of the complete dataset from the original experiment, the sample size was fixed at 20 participants, and no additional sample-size calculation was performed specifically for the upper-extremity outcomes.

### Apparatus

The experiment was conducted using two indoor force plates (Custom FP, Bertec Inc., Ohio, USA) and was surrounded by 13 motion-capture cameras (VICON MX, Vicon Motion Systems, Ltd., Oxford, UK). A motion capture system was used to record the body and ball motion at a sampling rate of 250 Hz. A set of 47 reflective markers was attached to the skin over specific anatomical landmarks according to a previous study [16]. Three reflective markers were attached to the index fingertip, middle fingertip, and interphalangeal joint of the middle finger. Furthermore, four reflective markers were attached to the ball. The ground reaction force (GRF) data were recorded at 1,000 Hz by two force plates and synchronized with the motion capture system. The global coordinate system (GCS) was a right-handed orthogonal coordinate system defined in a previous study [19].

### Experimental procedure

The participants pitched fastballs toward a target zone located 16 m away to examine pitching under different stride lengths. Initially, the individuals pitched five fastballs to a target zone with maximal effort, after a comfortable warm-up. For each participant, the stride length for the normal-stride condition (NS) was defined as the average stride length observed in the three fastest of the five initial baseline pitches. These baseline pitches were used only to determine the target stride length and were not included in subsequent analysis of ball speed, mechanical energy, or energy flow. The stride length was defined as the distance between the center of the ankle joint of the pivot leg at the maximal stride knee height and that of the stride leg at the stride foot contact. Based on the individually determined NS, two additional stride length conditions were established. The under-stride (US) and over-stride (OS) conditions were created by reducing or increasing the NS by 20%, respectively [19].

Visual reference lines were drawn on the force plate to guide stride foot placement, allowing participants to consistently pitch under each stride length condition (US, NS, or OS). We ensured participants had unlimited time to familiarize themselves with each stride length condition before measuring each condition. The participants pitched fastballs in each stride length condition until we collected ten balls that reached the target zone. Stride length conditions were tested in a randomized order, and experimental sessions were separated by one-week intervals to minimize learning and fatigue effects.

### Data analysis

Marker trajectories were filtered using a fourth-order zero-lag Butterworth low-pass filter [22]. The cutoff frequency was determined separately for each trial, marker, and coordinate according to a previously described procedure [23] and the resulting cutoff frequencies ranged from 6.2 to 20.3 Hz. The GRF data were filtered at 15 Hz to minimize artifacts in the computation of the joint moment [24,25] and subsequently resampled to 250 Hz using cubic spline interpolation to synchronize the marker data.

A whole-body model was developed using 16 rigid segments. Each segment and joint coordinate system definition followed the coordinate system used in baseball pitching studies [16,19]. The inertial parameters for each segment were estimated according to the definition of a previous study [26].

Mechanical energy was computed for the upper torso, upper arm, forearm, hand, and ball, and normalized by body mass. Intersegmental energy flow was quantified using the work of the joint force power (*JFP*), the segment torque power (*STP*), and the joint power (*JP*), which is the sum of the *JFP* and *STP*, by integrating each phase as described below. These quantities were evaluated at the trunk, shoulder, elbow, and wrist joints. The positive work represents the energy inflow to the adjacent distal segment through each joint, whereas the negative work indicates the energy outflow from each adjacent distal segment.

The pitching motion was separated into three phases based on three events as previously defined [3]. The stride phase was defined as the section between the maximal knee height (MKH) of the stride leg and the stride foot contact (SFC). The arm-cocking phase was between the SFC and the maximal external rotation (MER) of the throwing shoulder joint. The arm acceleration phase was between the MER and the ball release (REL). The SFC was defined as when the vertical GRF of the stride foot exceeded 5% of body mass [20]. The MER was identified as when reaching the maximum of humeral long-axis rotation relative to the thorax between SFC and REL, determined using a Z-Y’-Z” Euler rotation sequence. The REL was defined as when the distance between the center of the ball and the marker on the index fingertip exceeded 2 cm [27]. All data analyses were conducted using Python (version 3.11.7 for Windows).

### Statistical analysis

For each biomechanical variable, all 10 pitches that reached the target zone were included in the analysis. The values obtained from these 10 pitches were averaged within each participant and stride length condition, and these mean values were used for statistical analysis (S6 Table). Group data are presented as means and standard deviations (SDs). Participant was specified as the subject factor in all repeated measures analyses. Two-way repeated measures analysis of variance (ANOVA) was conducted to examine the effects of condition and event on mechanical energy and the effects of condition and phase on work, respectively. The assumption of sphericity was assessed using Mauchly’s test. When sphericity was violated (*p* < 0.05), Greenhouse–Geisser-adjusted degrees of freedom and *p* values were reported. A one-way repeated measures ANOVA was conducted to examine the effect of condition on ball speed. For the two-way repeated measures ANOVAs, when a significant condition × event or condition × phase interaction was observed, pairwise comparisons among stride length conditions were performed separately within each event or phase. For the one-way repeated measures ANOVA of ball speed, pairwise comparisons among stride length conditions were performed when a significant main effect of condition was observed. The *p* values and 95% confidence intervals (CIs) for the pairwise condition differences were adjusted using the Bonferroni correction. Partial eta squared (*η*^2^) was calculated to assess the effect size for main effects and interactions, where values of 0.01, 0.06, and 0.14 indicate small, medium, and large effects, respectively. Cohen’s *d* was calculated for post hoc pairwise comparisons, where values of 0.20, 0.50, and 0.80 indicate small, medium, and large effects, respectively. The significance level for all comparisons was *p*  <  0.05. All statistical analyses were performed using Python (version 3.11.7 for Windows).

## Results

Ball speeds obtained from the pitching trials analyzed in the present study have been reported previously [19]. The means and SDs of ball speed for the three stride-length conditions, together with the pairwise condition contrasts, are presented in Table 1 to provide context for the present upper extremity analysis. Ball speed was greater in the NS than in the US and OS, whereas no statistically significant difference was observed between the US and OS. These condition differences in ball speed provide the basis for examining how upper extremity energy flow differed among the stride length conditions.

**Table 1:** Ball speed across stride length conditions.

Descriptive statistics			One-way repeated measures ANOVA								Pairwise comparisons				
Variable	Condition	Mean ± SD (m/s)	Effect	Mauchly’s *W*	Mauchly’s *p*	Correction	df	*F*	*p*	*η²*	Comparison	*t*	95% CI (m/s)	*p*	*d*
Ball speed	US	32.49 ± 1.86	Condition	0.79	0.12	None	2.00, 38.00	10.99	< 0.01	0.36	NS–US	5.24	[0.71, 2.12]	0.03	0.784
	NS	33.90 ± 1.72									NS–OS	4.05	[0.50, 2.33]	0.03	0.791
	OS	32.49 ± 1.72									OS–US	0.01	[-1.08, 1.08]	1.00	0.001

US indicates the under-stride condition, NS indicates the normal-stride condition, and OS indicates the over-stride condition. Mauchly’s W and the corresponding p value indicate the results of Mauchly’s test of sphericity. Partial η2 indicates partial eta squared. CI indicates confidence interval. The p values and 95% CIs for pairwise condition comparisons were adjusted using the Bonferroni correction. d indicates Cohen’s d. Ball speed values were obtained from the same pitching trials reported previously [19].

S1 Table shows the results of two-way repeated measures ANOVAs for the mechanical energy at each segment. There were no significant differences in the mechanical energy of all segments and the ball across the stride length condition at MKH (Fig 1 and S2 Table). However, differences in the segmental mechanical energy were observed between conditions at SFC. The mechanical energy of the upper torso, upper arm, and forearm increased as stride length extended (Figs 1a – 1c, and S2 Table). The mechanical energy of the hand was greater in the NS than in the US, whereas no statistically significant differences were observed between the NS and OS or between the US and OS (Fig 1d and S2 Table). The mechanical energy of the ball was greater in the NS and OS than in the US, with no statistically significant difference between the NS and OS (Fig 1e and S2 Table). These relationships were not observed at MER. The mechanical energy of the upper torso at MER increased as stride length shortened (Fig 1a) and was particularly greater in the US than in the OS (*t* = 3.64, 95% CI (J/kg) = [0.03, 0.17], *p* = 0.01, *d* = 0.36). In contrast, the mechanical energy of the upper arm at MER was lower in the US than in the OS (*t* = 4.30, 95% CI (J/kg) = [0.04, 0.18], *p* < 0.01, *d* = 0.88) and NS (*t* = 5.51, 95% CI (J/kg) = [0.06, 0.16], *p* < 0.01, *d* = 0.80) (Fig 1b). Furthermore, the mechanical energy of the forearm, the hand, and the ball at MER was greater in the NS than in the OS (the forearm: *t* = 2.84, 95% CI (J/kg) = [0.004, 0.112], *p* = 0.03, *d* = 0.57, Fig 1c; the hand: *t* = 3.51, 95% CI (J/kg) = [0.02, 0.15], *p* = 0.01, *d* = 0.69, Fig 1d; the ball: *t* = 3.03, 95% CI (J/kg) = [0.01, 0.08], *p* = 0.02, *d* = 0.52, Fig 1e) and US (the forearm: *t* = 5.84, 95% CI (J/kg) = [0.06, 0.17], *p* < 0.01, *d* = 0.92, Fig 1c; the hand: *t* = 4.30, 95% CI (J/kg) = [0.03, 0.12], *p* < 0.01, *d* = 0.65, Fig 1d; the ball: *t* = 2.95, 95% CI (J/kg) = [0.003, 0.050], *p* = 0.02, *d* = 0.33, Fig 1e). A similar pa*t*tern was observed at REL, where the mechanical energy of the ball was greater in the NS than in the OS (*t* = 3.21, 95% CI (J/kg) = [0.01, 0.10], *p* = 0.01, *d* = 0.41) and US (*t* = 4.20, 95% CI (J/kg) = [0.03, 0.11], *p* < 0.01, *d* = 0.55) (Fig 1e). The pattern of condition differences in ball mechanical energy observed at REL was already present at MER.

**Fig 1:**
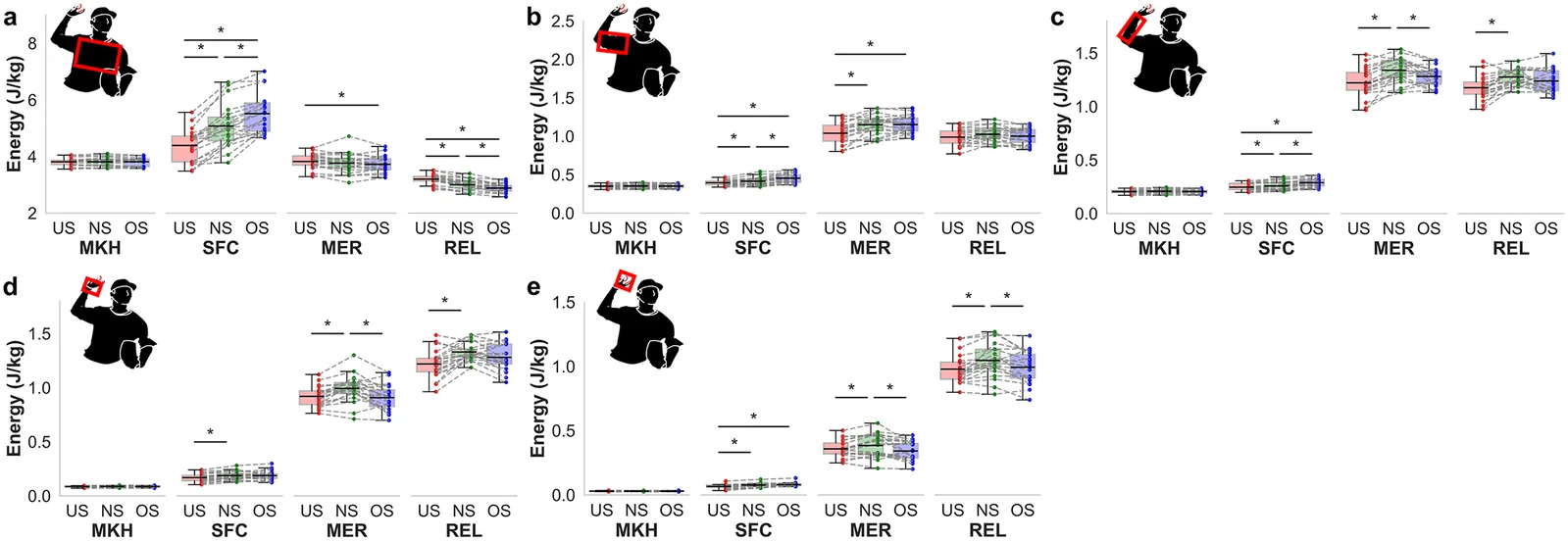
The mechanical energy at each event. a: The mechanical energy of the upper torso. b: The mechanical energy of the upper arm. c: The mechanical energy of the forearm. d: The mechanical energy of the hand. e: The mechanical energy of the ball. The box plot shows the interquartile range of the mechanical energy at each event. The black line in the box plot shows the mean mechanical energy at each event. The upper bar of the box plot shows the range from the third quartile to the maximum of the mechanical energy at each event, while the lower bar shows the range from the first quartile to the minimum. The dot plot shows the individual participant’s mechanical energy at each event. The dotted line shows the same participant’s mechanical energy for each stride length. The asterisk (*) shows a significant difference (*p*  <  0.05) across the stride length conditions.

We next examined energy flow between SFC and MER across body segments. S3 Table shows the results of two-way repeated measures ANOVAs for the energy flow at each joint. The *JP* at the trunk joint decreased and turned negative earlier as the stride length extended (Fig 2a). Therefore, the energy inflow to the upper torso through the trunk joint decreased as the stride length extended (Fig 3a and S4 Table). Additionally, the energy outflow from the upper torso through the trunk joint increased as the stride length extended (Fig 3b and S4 Table). The energy flow, the sum of the positive and negative integral *JP*, in the US was positive, while it was negative in the OS (Fig 3c and S4 Table). The *JP* at the shoulder joint was greatest in the OS at SFC (Fig 2b), as with the *JP* at the trunk joint. However, the peak value was higher in the NS because the rate of increase for *JP* was more gradual in the OS. As a result, the energy flow to the upper arm through the shoulder joint in the NS was greatest (NS-OS: *t* = 4.31, 95% CI (J/kg) = [0.11, 0.47], *p* < 0.01, *d* = 0.83; NS-US: *t* = 5.19, 95% CI (J/kg) = [0.15, 0.45], *p* < 0.01, *d* = 0.89; Fig 4c). On *t*he other hand, this value did not significantly differ between the US and OS (*t* = −0.07, 95% CI (J/kg) = [−0.27, 0.25], *p* = 1.00, *d* = 0.02). These relationships were shown at the elbow (Fig 4b) and wrist (Fig 4a) joints. The energy flow to the forearm through the elbow joint in the NS was greatest (NS-OS: *t* = 4.37, 95% CI (J/kg) = [0.09, 0.36], *p* < 0.01, *d* = 0.82; NS-US: *t* = 4.48, 95% CI (J/kg) = [0.07, 0.28], *p* < 0.01, *d* = 0.69), while the relationship be*t*ween the US and OS was not significantly different (*t* = 0.68, 95% CI (J/kg) = [−0.13, 0.22], *p* = 1.00, *d* = 0.18) (Fig 4b). The energy flow to the hand through the wrist joint in the NS was greatest (NS-OS: *t* = 4.12, 95% CI (J/kg) = [0.05, 0.23], *p* < 0.01, *d* = 0.68; NS-US: *t* = 2.95, 95% CI (J/kg) = [0.01, 0.14], *p* = 0.02, *d* = 0.37), while the relationship between the US and OS was not significantly different (*t* = 1.84, 95% CI (J/kg) = [−0.03, 0.16], *p* = 0.24, *d* = 0.38) (Fig 4a).

**Fig 2:**
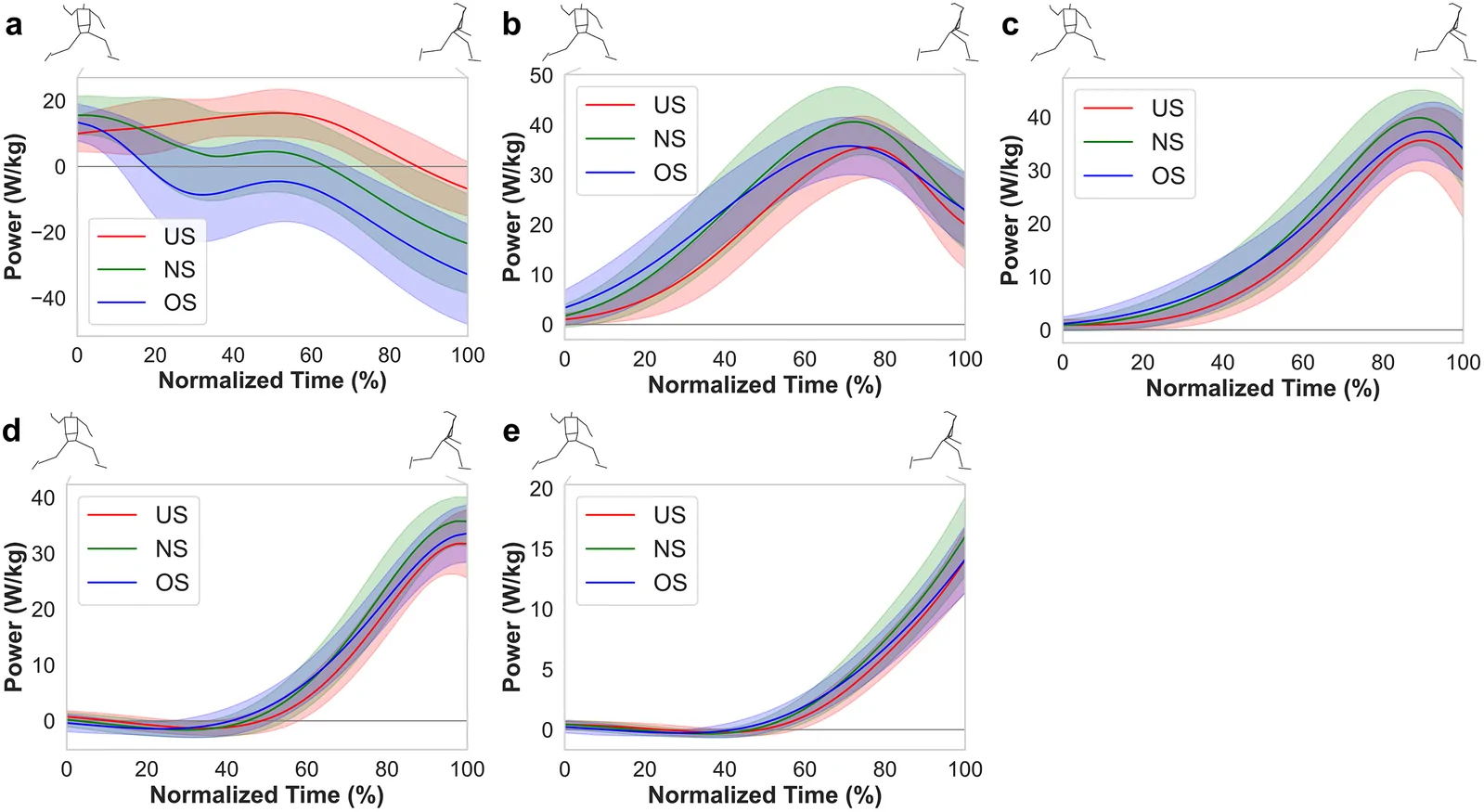
The *JP* during the arm-cocking phase. a: The *JP* at the trunk joint. b: The *JP* at the shoulder joint. c: The *JP* at the elbow joint. d: The *JP* at the wrist joint. The *JP* from SFC to MER was normalized from 0% to 100%. The thick line shows the mean *JP* for each stride length condition. The thin line shows the standard deviation of the *JP* for each stride length condition.

**Fig 3:**
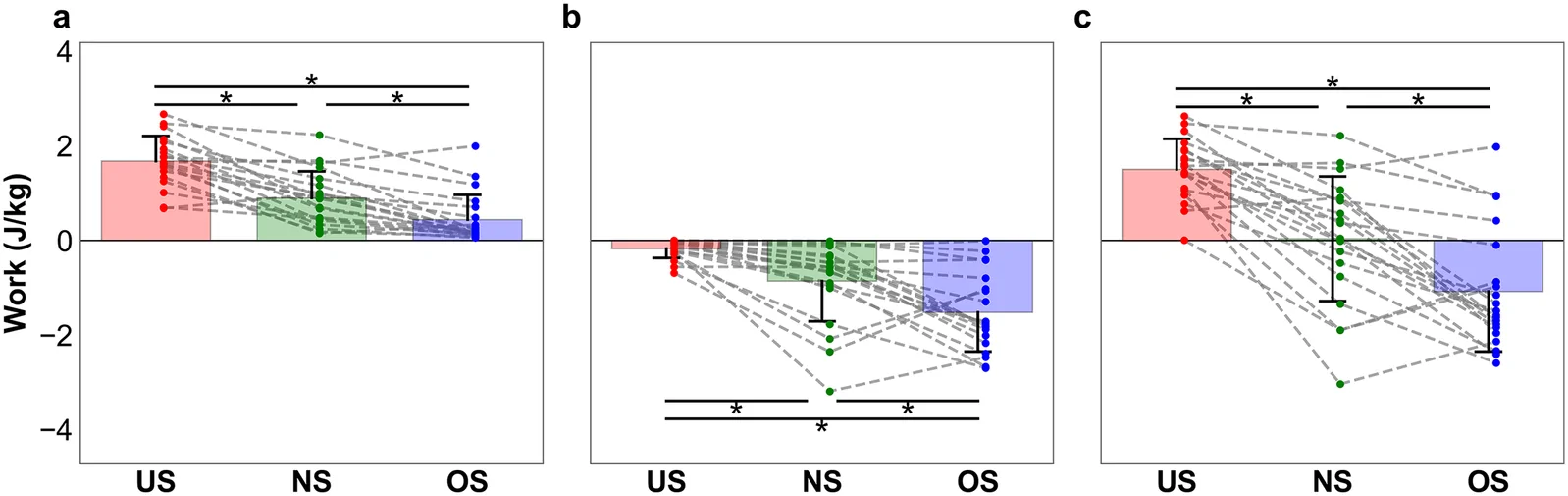
Energy flow to the upper torso through the trunk joint during the arm-cocking phase. a: The energy inflow to the upper torso through the trunk joint. b: The energy outflow from the upper torso through the trunk joint. c: The energy flow, the sum of the positive and negative integral *JP*, through the trunk joint. The bar shows the mean energy inflow, outflow, and flow during the arm-cocking phase for each stride length condition, respectively. The error bars show the standard deviation of energy inflow, outflow, and flow for each stride length condition, respectively. The dot plots show the individual participant’s energy inflow, outflow, and flow for each stride length condition, respectively. The dotted line shows the same participant’s energy inflow, outflow, and flow for each stride length condition, respectively. The asterisk (*) shows a significant difference (*p*  <  0.05) across the stride length conditions.

**Fig 4:**
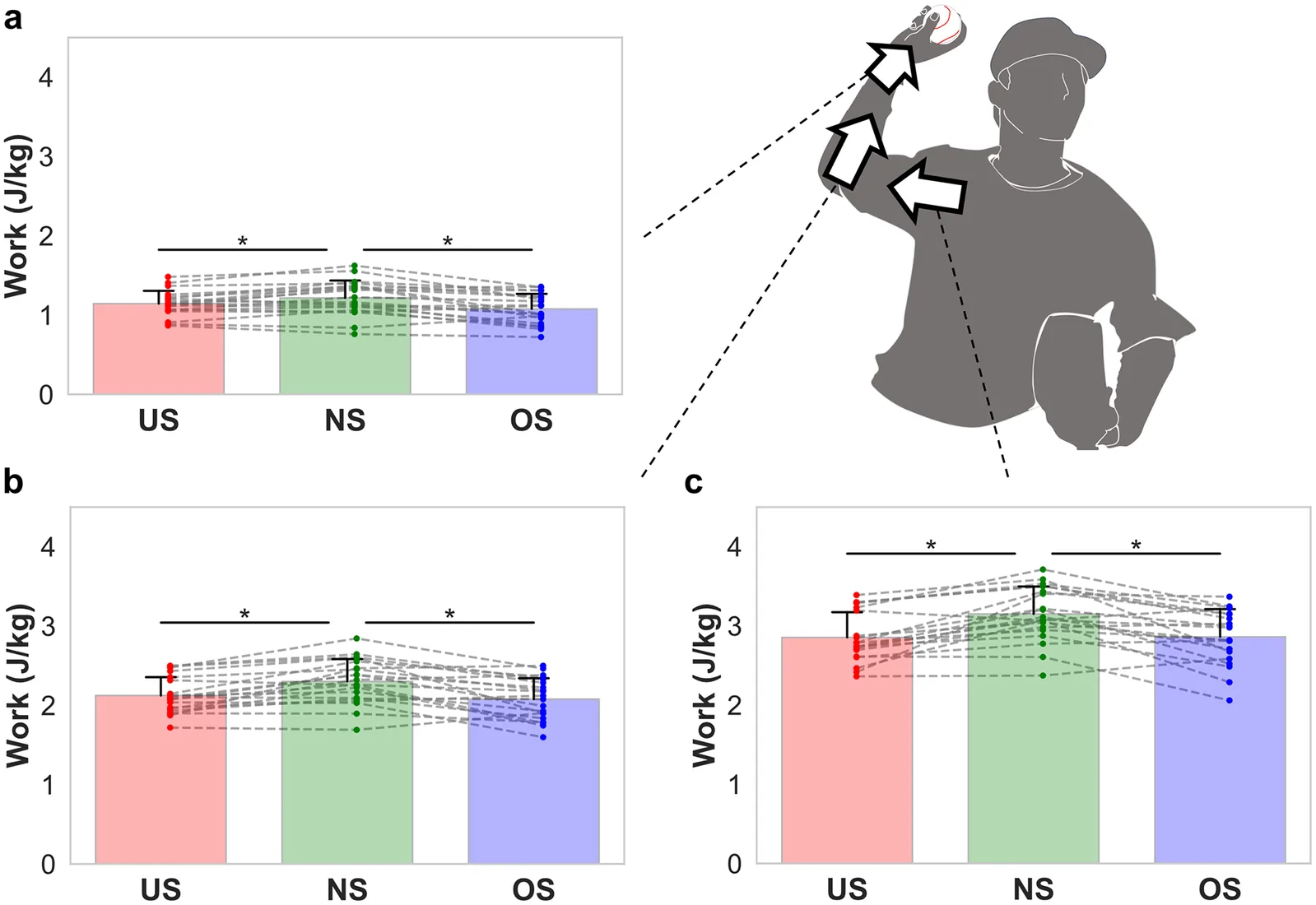
Energy flow through each joint during the arm-cocking phase. a: The energy flow to the hand through the wrist joint. b: The energy flow to the forearm through the elbow joint. c: The energy flow to the upper arm through the shoulder joint. The bar shows the mean energy flow during the arm-cocking phase for each stride length condition. The error bars show the standard deviation of energy flow for each stride length condition. The dot plots show the individual participant’s energy flow for each stride length condition. The dotted line shows the same participant’s energy flow for each stride length condition. The asterisk (*) shows a significant difference (*p*  <  0.05) across the stride length conditions.

We further examined whether the components of energy flow at the upper limb during the arm-cocking phase were different among stride length conditions. S5 Table shows the results of two-way repeated measures ANOVAs for the energy flow originating from *JFP* and *STP* at each joint. At the shoulder joint, the energy flow originating from the *JFP* was greater in the NS than in the US (*t* = 6.13, 95% CI (J/kg) = [0.17, 0.41], *p* < 0.01, *d* = 0.93) and OS (*t* = 3.11, 95% CI (J/kg) = [0.02, 0.28], *p* = 0.02, *d* = 0.55), whereas there was no significant difference between the US and OS (*t* = −1.89, 95% CI (J/kg) = [−0.33, 0.05], *p* = 0.22, *d* = 0.49) (Fig 5c). The same pattern was observed at the elbow (Fig 5b) and wrist (Fig 5a) joints. At the elbow joint, the energy flow originating from the *JFP* was greater in the NS than in the US (*t* = 4.35, 95% CI (J/kg) = [0.10, 0.40], *p* < 0.01, *d* = 0.63) and OS (*t* = 4.75, 95% CI (J/kg) = [0.14, 0.50], *p* < 0.01, *d* = 0.86), whereas there was no significant difference between the US and OS (*t* = 0.83, 95% CI (J/kg) = [−0.16, 0.31], *p* = 1.00, *d* = 0.21) (Fig 5b). At the wrist joint, the energy flow originating from the *JFP* was greater in the NS than in the US (*t* = 3.24, 95% CI (J/kg) = [0.02, 0.17], *p* = 0.01, *d* = 0.40) and OS (*t* = 3.69, 95% CI (J/kg) = [0.04, 0.25], *p* < 0.01, *d* = 0.58), whereas there was no significant difference between the US and OS (*t* = 1.26, 95% CI (J/kg) = [−0.06, 0.16], *p* = 0.66, *d* = 0.24) (Fig 5a). In contrast, the energy flow originating from the *STP* at the shoulder joint was lower in the OS than in the US (*t* = 3.98, 95% CI (J/kg) = [0.04, 0.22], *p* < 0.01, *d* = 0.75) and NS (*t* = 5.57, 95% CI (J/kg) = [0.07, 0.20], *p* < 0.01, *d* = 0.76) (Fig 6c). The energy flow originating from the *STP* at the elbow joint showed negative values in all stride length conditions, indicating energy outflow from the forearm through the elbow joint. These values were lower in the NS than in the US (*t* = −2.77, 95% CI (J/kg) = [−0.142, −0.004], *p* = 0.04, *d* = 0.34) and OS (*t* = −3.96, 95% CI (J/kg) = [−0.17, −0.03], *p* < 0.01, *d* = 0.53), whereas there was no significant difference between the US and OS (*t* = −1.02, 95% CI (J/kg) = [−0.10, 0.05], *p* = 0.97, *d* = 0.16) (Fig 6b). The energy flow originating from the STP at the wrist joint was nearly zero across all stride length conditions (Fig 6a). Neither a statistically significant main effect of condition nor a phase × condition interaction was observed (S5 Table).

**Fig 5:**
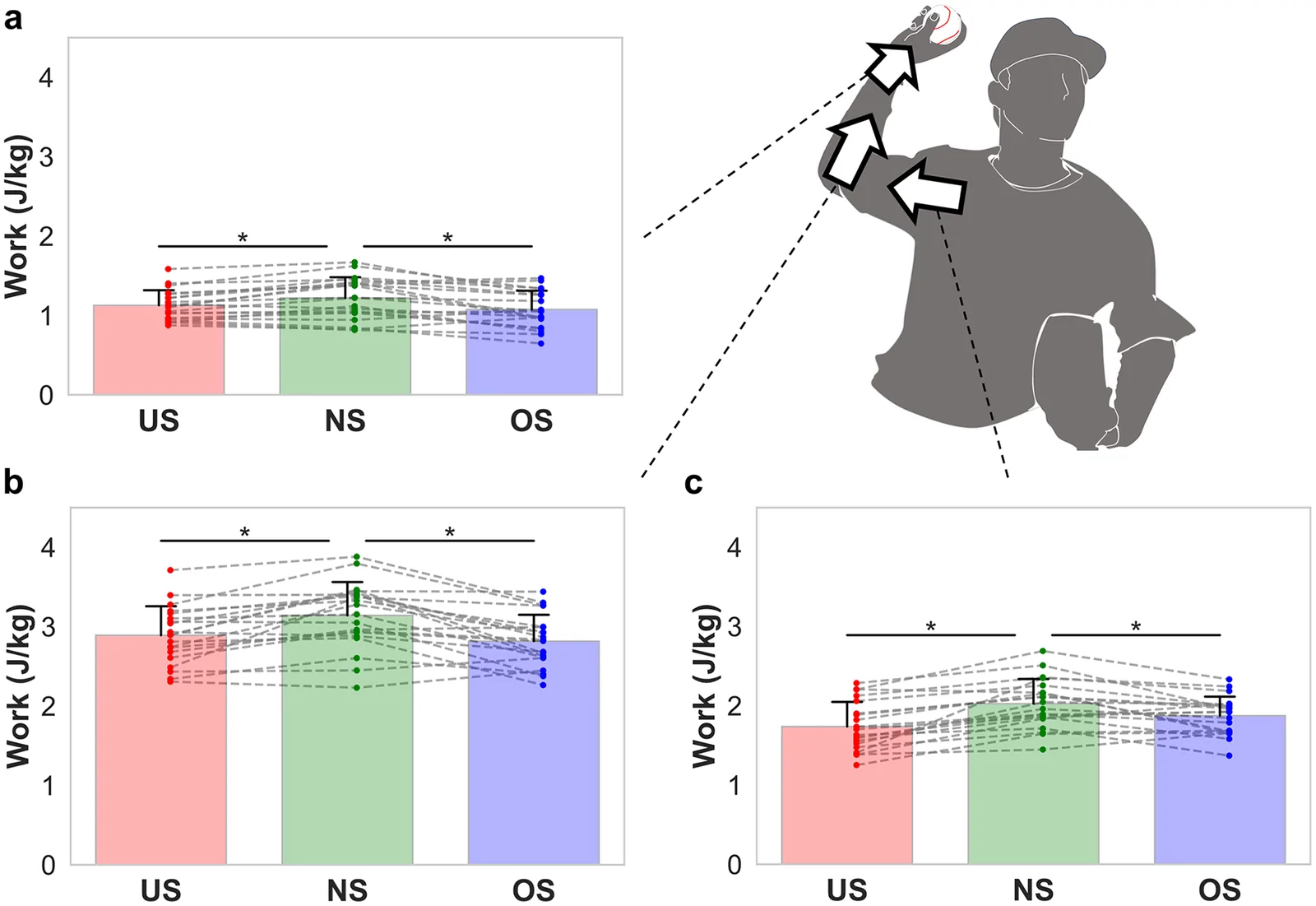
The energy flow originating from the *JFP* during the arm-cocking phase. a: The energy flow originating from the *JFP* to the hand through the wrist joint. b: The energy flow originating from the *JFP* to the forearm through the elbow joint. c: The energy flow originating from the *JFP* to the upper arm through the shoulder joint. The bar shows the mean energy flow originating from the *JFP* during the arm-cocking phase for each stride length condition. The error bars show the standard deviation of energy flow originating from the *JFP* for each stride length condition. The dot plots show the individual participant’s energy flow originating from the *JFP* for each stride length condition. The dotted line shows the same participant’s energy flow originating from the *JFP* for each stride length condition. The asterisk (*) shows a significant difference (*p*  <  0.05) across the stride length conditions.

**Fig 6:**
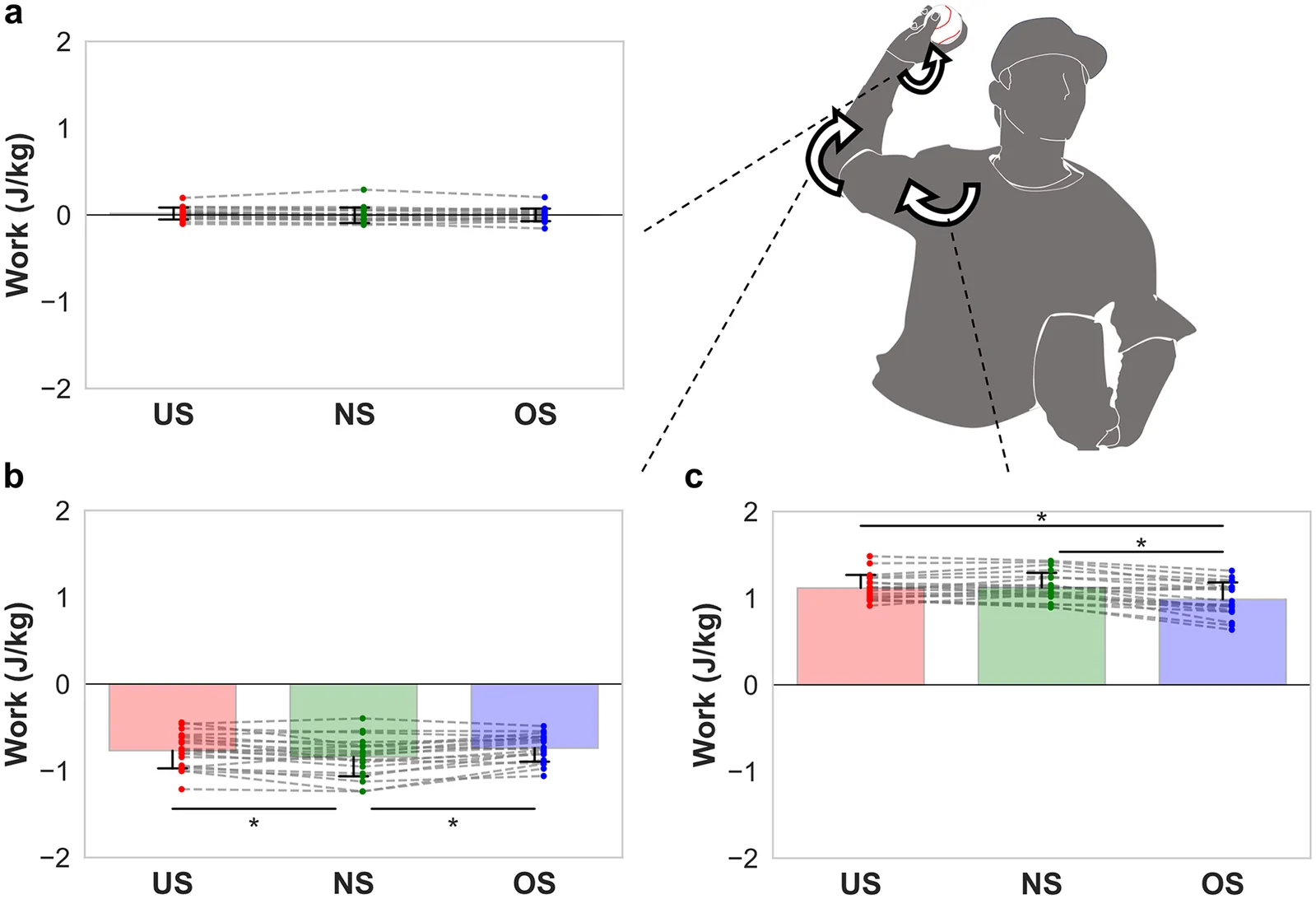
The energy flow originating from the *STP* during the arm-cocking phase. a: The energy flow originating from the *STP* to the hand through the wrist joint. b: The energy flow originating from the *STP* to the forearm through the elbow joint. c: The energy flow originating from the *STP* to the upper arm through the shoulder joint. The bar shows the mean energy flow originating from the *STP* during the arm-cocking phase for each stride length condition. The error bars show the standard deviation of energy flow originating from the *STP* for each stride length condition. The dot plots show the individual participant’s energy flow originating from the *STP* for each stride length condition. The dotted line shows the same participant’s energy flow originating from the *STP* for each stride length condition. The asterisk (*) shows a significant difference (*p*  <  0.05) across the stride length conditions.

## Discussion

This study investigated how upper extremity energy flow differed across stride length conditions in relation to the observed differences in ball speed. The main results showed that the energy flow to the upper arm through the shoulder joint during the arm-cocking phase was greater in the NS than in the US and OS. Furthermore, the composition of *JFP* and *STP* in the energy flow to the upper arm was different, while the energy flow to the upper arm was not statistically significantly different between US and OS.

The mechanical energy of the upper torso, the upper arm, the forearm, the hand, and the ball in pitching changed as the stride length altered (Fig 1). The mechanical energy of the proximal segments, particularly the upper torso and the upper arm, increased as the stride length extended at the SFC (Figs 1a and 1b). These findings are consistent with previously reported patterns of mechanical energy in the lower torso [19], indicating that the mechanical energy in proximal segments increased during the stride phase as the stride length extended. If the energy flow to the distal segments were similar to that of the proximal segment, the difference in the mechanical energy of the distal segments at the MER among stride length conditions would be the same as that of the proximal segments at the SFC. However, the mechanical energy of the distal segments, particularly the forearm and the hand, and the ball at the MER was higher in the NS than in the US and OS (Figs 1c - 1e), suggesting that altering stride length changes the amount of energy flow to the upper limbs between the SFC and MER, which is the arm-cocking phase.

We then examined energy flow between body segments to reveal how it differs across stride length conditions during the arm-cocking phase. The *JP* at the trunk joint transitioned from positive to negative earlier as the stride length extended (Fig 2a). Therefore, the energy outflow from the upper torso through the trunk joint increased as the stride length extended (Fig 3b). On the other hand, as the stride length shortened, the positive duration of the *JP* increased, leading to greater energy inflow to the upper torso through the trunk joint (Fig 3a). As a result, the energy flow, the integral of the sum of the positive and negative *JP*, to the upper torso through the trunk joint showed a positive value in the US and a negative value in the OS (Fig 3c). Considering our findings that the mechanical energy from the MKH to SFC increased as the stride length extended (Fig 1a), our results indicate that a longer stride length facilitates earlier energy inflow to the upper torso while simultaneously increasing the energy outflow from the upper torso to the lower torso. A previous study reported that the energy outflow from the lower torso to the stride hip joint and energy absorption at the stride hip joint increased during the arm-cocking phase as the stride length extended [19]. These findings suggest that extending stride length allows for earlier energy inflow to the upper torso while simultaneously increasing the energy outflow from the upper torso to the stride leg.

The energy flow through the shoulder, elbow, and wrist joints was greater in the NS than in the US and OS (Fig 4). The elbow and wrist joints primarily transfer energy from proximal to distal segments and generate little mechanical energy [16,18,28]. In this study, the energy flow originating from *JFP*, representing energy transfer, at the elbow and wrist joints showed a similar trend to that observed at the shoulder joint, where the NS was greater than the OS and US (Figs 5a and 5b). In contrast, the energy flow originating from *STP,* which may reflect both energy generation and transfer, showed a negative value at the elbow joint across all stride length conditions (Fig 6b) and was nearly zero at the wrist joint (Fig 6a). These findings indicate that energy was primarily transferred from the upper arm to the hand, while the contribution of STP to energy flow toward the distal segments was limited at the elbow and wrist joints. Previous studies have reported associations between energy flow to the upper arm and ball speed. Howenstein et al. (2019) reported that the energy flow to the upper arm showed the strongest correlation with the ball speed in youth baseball pitchers. Furthermore, Wasserberger et al. (2021) reported that energy transfer from the torso to the upper arm during the arm-cocking phase accounted for an additional 24.6% of the variance in pitch speed beyond body mass and height. The present study showed that the difference in ball speed across stride length conditions was consistent with the difference in energy flow to the upper arm (Fig 4c) and its transfer to the forearm and hand (Figs 5a and 5b). Taken together, these previous findings and the present results raise the possibility that energy flow to the upper arm through the shoulder sets the amount subsequently transferred distally and, ultimately, ball speed, even as lower-extremity mechanics vary. Further analyses accounting for the serial transfer of energy across segments are needed to test this possibility, as discussed in the Limitations section.

The US and OS conditions showed no statistically significant difference in ball speed despite a difference in the composition of shoulder energy flow. Previous studies have suggested that different stride lengths can produce distinct mechanical strategies without a statistically significant difference in ball speed. Specifically, an extending stride length pitch increased total body linear momentum [14], whereas the shortening stride length pitch increased transverse-plane angular momentum [15]. In the present study, no statistically significant differences in total energy flow from the shoulder joint onward were observed between the US and OS conditions. Taken together, these findings raise the possibility that distinct lower extremity mechanics may be accompanied by differences in shoulder energy flow composition, defined in the present study as the energy flow originating from *JFP* and that originating from *STP*, even when no statistically significant difference is observed in the total energy flow into the upper arm through the shoulder joint. Our results showed that the energy flow originating from *STP* to the upper arm through the shoulder joint was higher in the US than in the OS, whereas no statistically significant difference in the energy flow originating from JFP was observed between these conditions (Figs 5c and 6c). These results further suggest that shoulder energy flow composition may differ between stride length conditions even when no statistically significant difference is observed in the total energy flow to the upper arm.

These findings imply that achieving higher ball speed may not depend solely on increasing the total energy flow into the upper arm through the shoulder joint, but also on how that energy is delivered. Specifically, an effective combination of energy transfer and energy generation, without reducing either component, may contribute to greater energy delivery to the distal segments. Notably, the NS showed greater energy flow originating from the *JFP* without a reduction in energy originating from the *STP* (Figs 5c and 6c). This perspective highlights the potential importance of considering not only the magnitude but also the composition of energy flow when evaluating pitching performance. However, the optimal composition of energy flow to increase ball speed remains unclear and warrants further investigation.

## Limitations

This study had some limitations. First, the stride length conditions were individualized relative to the NS condition because within-pitcher manipulations of pitching mechanics are most meaningfully referenced to each pitcher’s self-selected motion rather than to an externally imposed absolute stride length. This approach is consistent with previous studies [15,20], which manipulated stride length relative to each pitcher’s stride length. However, because the NS was derived from the three baseline pitches with the highest ball speeds, the higher ball speed observed in the NS condition may partly reflect the procedure used to define the reference condition.

Second, as acknowledged in our previous report based on the same pitching trials [19], all pitches were performed on flat ground rather than from a regulation pitching mound. Previous studies have reported differences in ball speed, pitching mechanics, and upper extremity joint loading between flat ground throwing and pitching from a mound [30–32]. These differences may also affect the magnitude and composition of energy flow through the upper extremity. Therefore, the present findings may not be directly generalizable to pitching from a regulation mound.

Third, the present study focused on the energy transmitted into the adjacent distal segment at each upper extremity joint because it aimed to determine how mechanical energy is transmitted through the throwing arm to the ball. This approach enabled us to examine energy flow along the proximal-to-distal pathway toward the ball but did not account for all energy flowing into and out of each segment. A complete account of segmental energy flow would require consideration of the power acting at both the proximal and distal ends of each segment and, where applicable, at all joints connected to that segment [29,33]. Therefore, the reported values should be interpreted as energy flow along the selected pathway toward the ball rather than as a complete account of the energy entering and leaving each segment.

Fourth, the energy flow analysis used in the present study quantified energy transmitted into adjacent distal segments but could not quantify how mechanical energy is distributed among non-adjacent segments from dynamic coupling. Consequently, the present study could not clarify how the mechanical energy entering the upper arm through the shoulder joint is subsequently transferred distally and ultimately contributes to ball speed. Previous multibody energetic analyses have decomposed the generation and redistribution of mechanical energy into muscular and interaction-related contributions during baseball pitching [18]. Induced acceleration analysis has also been used to quantify how proximal joint torques and velocity-dependent torques contribute to distal joint accelerations during throwing [28], but it does not quantify mechanical energy transfer. Although induced power analysis extends this decomposition to power [34], it does not directly identify the source or pathway of mechanical energy transfer across non-adjacent segments. Further methodological development is therefore needed to quantify such transfer within a multi-segment system while accounting for dynamic coupling.

## Conclusion

We found that altering the stride length changed the timing and amount of energy flow through the trunk joint during the arm-cocking phase. We also observed that the energy flow into the upper arm through the shoulder joint was greater in the NS than in the US and OS during the arm-cocking phase. The NS also showed greater energy transfer through the elbow and wrist joints and greater ball speed than the US and OS. These findings raise the possibility that energy flow into the upper arm through the shoulder joint sets the amount of energy subsequently transferred distally and, ultimately, ball speed. Moreover, the US and OS conditions suggest that altering lower extremity mechanics may alter shoulder energy flow composition even when no statistically significant difference is observed in total energy flow into the upper arm through the shoulder joint.

## Supporting information

S1 Table Two-way repeated measures ANOVA results for mechanical energy. Mauchly’s *W* and the corresponding *p* value indicate the results of Mauchly’s test of sphericity. Greenhouse–Geisser-adjusted degrees of freedom and *p* values are reported when sphericity was violated. Partial *η²* indicates partial eta squared. *ε* indicates the Greenhouse–Geisser epsilon.
(XLSX)

S2 Table Pairwise condition contrasts in mechanical energy at MKH and SFC. MKH indicates maximal knee height, and SFC indicates stride foot contact. CI indicates confidence interval. *p* values and 95% CIs were adjusted using the Bonferroni correction. d indicates Cohen’s *d*.
(XLSX)

S3 Table Two-way repeated measures ANOVA results for energy flow. Energy flow was quantified from joint power (*JP*). Mauchly’s *W* and the corresponding *p* value indicate the results of Mauchly’s test of sphericity. Greenhouse–Geisser-adjusted degrees of freedom and *p* values are reported when sphericity was violated. Partial *η²* indicates partial eta squared. *ε* indicates the Greenhouse–Geisser epsilon.
(XLSX)

S4 Table Pairwise condition contrasts in trunk joint energy flow into the upper torso during the arm-cocking phase. CI indicates confidence interval. *p* values and 95% CIs were adjusted using the Bonferroni correction. *d* indicates Cohen’s *d*.
(XLSX)

S5 Table Two-way repeated measures ANOVA results for energy flow originating from the *JFP* and *STP* at upper limb joints. *JFP* indicates joint force power, and *STP* indicates segment torque power. Mauchly’s *W* and the corresponding *p* value indicate the results of Mauchly’s test of sphericity. Greenhouse–Geisser-adjusted degrees of freedom and *p* values are reported when sphericity was violated. Partial *η²* indicates partial eta squared. *ε* indicates the Greenhouse–Geisser epsilon.
(XLSX)

S6 Table Trial data underlying the statistical analyses reported in the present study. (XLSX)
